# Optimization of Forced Degradation Using Experimental Design and Development of a Stability-Indicating Liquid Chromatographic Assay Method for Rebamipide in Bulk and Tablet Dosage Form

**DOI:** 10.3797/scipharm.1011-06

**Published:** 2011-01-10

**Authors:** Sandeep Sonawane, Paraag Gide

**Affiliations:** MET’s Institute of Pharmacy, MET League of Colleges, Bhujbal Knowledge City, Nashik 422 003, Maharashtra State, India

**Keywords:** Experimental design, Rebamipide, Validation, Stability-indicating, Microwave energy, RP-HPLC, Degradation product

## Abstract

A novel stability-indicating RP-HPLC assay method was developed and validated for quantitative determination of rebamipide in bulk and tablet dosage form. Rebamipide (drug and drug product) solutions were exposed to acid and alkali hydrolysis, thermal stress, oxidation by hydrogen peroxide and photodegradation. Experimental design has been used during forced degradation to determine significant factors responsible for degradation and to obtain optimal degradation conditions. In addition, acid and alkali hydrolysis was performed using a microwave oven. The chromatographic method employed the HiQ sil C-18HS (250 × 4.6 mm; 5 μm) column with mobile phase consisting of 0.02 M potassium phosphate (pH adjusted to 6.8) and methanol (40:60, *v/v*) and the detection was performed at 230 nm. The procedure was validated for specificity, linearity, accuracy, precision and robustness. There was no interference observed of excipients and degradation products in the determination of the active pharmaceutical ingredient. The method showed good accuracy and precision (intra and inter day) and the response was linear in a range from 0.5 to 5 μg mL^−1^. The method was found to be simple and fast with less trial and error experimentation by making use of experimental design. Also, it proved that microwave energy can be used to expedite hydrolysis of rebamipide.

## Introduction

Forced degradation is an integral part of drug development process. Although most of the literature defines the concept of forced degradation they do not provide detailed information about a forced degradation strategy [1, 2]. The experimental conditions to conduct forced degradation are described in a general way [3, 4] but exact stress conditions to be applied are not described.

Generally, a trial and error approach is adopted to select the strength, temperature and time of exposure to achieve degradation to an extent of 10–20% [5]. Such trial and error approach is cost, time and labor intensive and should be substituted by more systematic approaches. Also, if the experiments are performed randomly the results obtained will also be random. Therefore, it is necessary to plan the experiments in such a way that the real experimental situation as well as the theoretical background of experiments is obtained.

Factorial design is a type of experimental design in which all the possible combinations of factors and levels are investigated [6, 7]. Also, it has been observed that the conventional method of heating the drug in presence of water, acid or alkali using a heating mantle or water bath can prove to be time consuming and a comparatively slow process, ich can be substituted by use of microwave energy. The conventional method of heating uses an external source, in which the heat transfer depends upon the thermal conductivity of various materials. This heat initially leads to the increase in the temperature of the reaction vessel and thereafter, of the reaction mixture. By contrast, microwave energy produces efficient internal heating by direct coupling of microwave energy with polar molecules. Microwave assisted reactions are mainly based on the efficient heating of materials by microwave dielectric heating effects. Microwave dielectric heating depends on the ability of a specific material to absorb microwave energy and convert it into heat. Hence it is less time consuming [8].

**Fig. 1.**
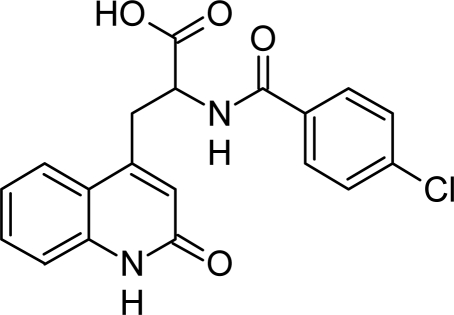
Chemical Structure of Rebamipide

Chemically, Rebamipide is *N*-[(4-chlorophenyl)carbonyl]-3-(2-oxo-1,2-dihydroquinolin-4-yl)alanine (Fig. 1) [9].

It is a fine white to off-white powder that is soluble in dimethyl formamide, sparingly soluble in methanol, water, ethyl acetate and acetonitrile, and practically insoluble in *n*–hexane. It is an antiulcer agent used for mucosal protection, healing of gastro duodenal ulcers and in the treatment of gastritis [10]. It is also used for the treatment of Behcet’s disease [11]. Rebamipide is known to be rapidly absorbed following oral application (typical dose is 100 mg).

Literature survey revealed very few quantitative analytical methods for estimation of rebamipide with bioanalytical applications [12–15]. Till date no stability-indicating HPLC method has been reported for quantification of rebamipide

The ICH guidelines require performing forced degradation of drug substance, which can help to indentify the likely degradation products and can be useful to establish degradation pathways and validate a stability-indicating method. Moreover, the validated stability-indicating method should be applied to the stability-study. Thus, the aim of this study was to develop and validate stability-indicating LC method for quantification of rebamipide in bulk and tablets according to official codes. Also, an attempt was made to simplify forced degradation studies by adopting experimental design and microwave energy to expedite hydrolysis.

## Materials and Methods

### Drug and Chemicals

Pharmaceutical grade rebamipide was procured as gift sample from Macleods Pharmaceuticals Ltd., Mumbai (India), certified to contain 99.96 % and used without further purification. Methanol and water used in analysis were of HPLC grade. Potassium dihydrogen phosphate, dipotassium hydrogen phosphate, hydrochloric acid, sodium hydroxide, hydrogen peroxide used were of analytical grades. All chemicals were purchased from SD Fine Chemicals, Mumbai (India). The 0.45 μ Nylon filter papers were purchased from Pall India Pvt. Ltd, Mumbai (India).

### Equipments:

HPLC system used consisted of pump PU – 2080 plus (JASCO, Japan) with Rheodyne loop injector (7725*i*) of capacity 20 μL. Detection was carried out by UV – 2075 detector (JASCO, Japan). The data acquisition was done using Borwin chromatography software version 1.50.

Acid and alkali induced forced degradation was carried out using Catalyst Scientific Microwave Oven System (Model: CATA 2R, Range: 140–700 W, Make: Catalyst System, Pune, India).

### Chromatographic Conditions

The chromatographic separation was performed in an HiQ sil C-18HS column (250 × 4.6 mm I. D., 5 μm, Kya Technologies Corporation, JAPAN). The mobile phase comprising a mixture of 0.02 M potassium phosphate (pH adjusted to 6.8) and methanol (40:60, *v/v*) at a flow-rate of 1.0 mL. min^−1^ with iscocratic elution. The injection volume was 20 μL for both reference substance and drug product solutions and the run time was 7 min. Detection was carried out at 230 nm.

## Experimental

### Forced Degradation Study Generation of Forced Degradation Samples

The forced degradation study was conducted on rebamipide in both bulk and tablets as well as on placebo (excipients excluding drug). Tablets containing 100 mg of rebamipide, were prepared in-house.

All stress studies were performed at an initial drug concentration of 1 mg.mL^−1^. Solutions of rebamipide tablets in each medium were prepared by weighing an amount of finely powdered tablets equivalent to an amount 100 mg of rebamipide. The solubilization of the drug from tablet powder in each medium was effected by sonicating it for 10 min followed by filtration and the final volume was adjusted with methanol.

Acidic and alkaline hydrolysis were performed by using microwave oven with full factorial optimization procedure. On the basis of preliminary experiments three independent factors; strength of acid/alkali (Normality), irradiation time (min) and microwave power (Watt) were chosen as input and % degradation as output. Similarly, to perform degradation under wet heat conditions using microwave oven, two factors were considered; irradiation time (min) and microwave power (Watt) as input and % degradation as output.

The high level of each factor was considered as “+1” and low level as “−1”.

Oxidative studies were carried out at room temperature in 30 % hydrogen peroxide for 24h then heated on water bath for 10 min to remove excess of hydrogen peroxide.

At the end of exposure, the samples from each hydrolyzed and oxidized drug solutions were diluted to 100 mL with methanol. The flasks were thoroughly covered with aluminium foil, labeled appropriately and stored in refrigerator till analysis.

The photodegradation study was performed by exposing the drug powder, spread as a thin film in a covered petri plates and exposed to direct sunlight for 48 h. Similarly, tablets of rebamipide was taken in petri plates and exposed to direct sunlight for 48 h. A control study in dark was also run simultaneously.

For the dry heat study, the drug powder and tablets of rebamipide were taken in separate petri plates and kept in oven at 80 °C for 48 h.

Solutions of photodegraded and dry heat condition samples of drug powder were prepared by dissolving 100 mg of sample in sufficient methanol to produce 100 mL. While, from photodegraded tablets and tablets exposed to dry heat in an oven; powder equivalent to 100 mg of rebamipide was weighed and dissolved in sufficient methanol, sonicated for 10 min, filtered and volume was made to 100 mL with methanol. Finally, each sample solution was diluted with mobile phase to get final concentration of 10 μg mL^−1^ and subjected to chromatographic analysis. In each case, suitable blanks and controls were used to preclude errors.

### Method Validation

Validation was performed with respect to ICH guideline Q2 (R1) [16]. To establish linearity and range; standard stock solution containing 1mg mL^−1^ of rebamipide in methanol was diluted with mobile phase to get solutions in the concentration range of 0.5–5 μg mL^−1^. The calibration curve standards were analyzed in triplicate and the peak area was plotted against corresponding drug concentration and subjected to least square linear regression to get an equation for best fit line.

To perform formulation assay; 20 tablets were weighed and finely powdered. A quantity of powder equivalent to 100 mg of rebamipide was transferred to 100 mL volumetric flask and sonicated with 70 mL methanol for 10 min, filtered and volume was made up to the mark with methanol. Similarly, the placebo stock solution was prepared. Suitable dilutions were made with mobile phase and subjected to chromatographic analysis.

The accuracy and precision were determined by fortifying a placebo with 80 mg, 100 mg and 120 mg of rebamipide (80 %, 100 % and 120 % of the label claimed, respectively) diluted suitably with mobile phase. The resulting mixtures were analyzed in triplicates over three days. The % recovery of added drug and % RSD were taken as a measure of accuracy and precision, respectively. Also, the results obtained were subjected to one way ANOVA and within-day mean square and between-day mean square was determined and compared using F-test.

For the determination of specificity, a placebo mixture was subjected to chromatographic analysis before and after applying stress conditions. Absence of peaks in the blank runs at the retention time of drug was taken as indication of specificity.

## Results and Discussion

From the preliminary experiments no degradation was found under acid and water hydrolysis using microwave oven as well as with conventional method of heating. Hence, it was decided not to proceed further with these conditions. But for alkali hydrolysis with microwave oven as well as under conventional method of heating, degradation has been obtained. Hence, it was decided to apply an experimental design for it to get an optimum degradation conditions.

Three independent factors (concentration of alkali, irradiation time and microwave power) were studied at two levels (−1 and +1) i.e. 2^3^ factorial design. Hence, 8 experiments were performed. The experimental matrix and resulting % degradation are presented in Table 1.

From the Table 1, the relationship obtained between input and output was evaluated by using multiple regression equation;

Y=β0+β1X1+β2X2+β3X3+β12X1X2+β13X1X3+B23X2X3+β123X1X2X3

where, Y is the output (% degradation), β0 is the intercept, β1, β2, β3, β12, β23, β13 and β123 represents regression coefficients and X1, X2 and X3 as independent variables.

The values of regression coefficient (βi) for multiple regression equation were calculated as;

$$\beta i=\frac{\sum Xi}{2^{n}}$$

where, Xi is the value of column (X1, X2 or X3) and Y is the % degradation obtained. From the coefficient obtained the multiple regression equation for alkaline degradation was obtained as;

Eq. 1. Y=13.62+1.88 X1+4.12 X2+11.12 X3+0.88 X1X2+1.62 X2X3+0.38 X1X3−0.62 X1X2X3

Further, to determine the significant factors with respect to Y (% degradation); Yates analysis was performed and the F values were determined. It has been found that the F-value for irradiation time and in combination with microwave power has significant effect on % degradation of rebamipide. Therefore, the use of these factors for determination of % degradation in the optimization design was justified.

Also, the magnitude of corresponding coefficients in multiple regression equation shows their significant effect on each factor. Thus, from the Eq. 1, it has been observed that factor X3 (microwave power) has more significant effect on % degradation of rebamipide than factor X2 (irradiation time) and factor X1 (concentration of NaOH).

Thus, by substituting X1 = 0 in Eq. 1; the new equation obtained is;

Eq. 2. Y=13.62+4.12 X2+11.12 X3+1.62 X2X3

By rearranging the Eq.2,

$$X3=\frac{\text{Y}-13.62-4.12\;\text{X}2}{11.12\;\text{X}1+1.62\;\text{X}2}$$

**Fig. 2.**
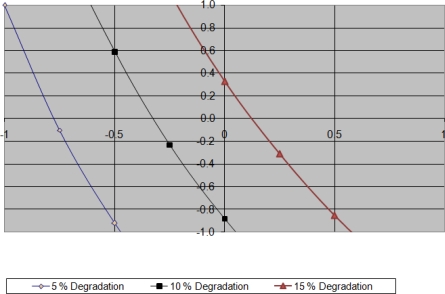
Contour Plot for Alkali degradation

Further, the values of Y (% degradation) were assumed to be 5, 10 and 15 %. Thus, the values for X3 at various levels of X2 such as X2 = −1, −0.75, −0.5, −0.25, 0, 0.25, 0.5, 0.75 and 1 were calculated and contour plot was plotted as shown in Fig2.

To get actual values for three factors, the coded values were decoded as follows;

$$\text{Transformed}\;\text{Value}=\frac{\text{X}-\text{the}\;\text{average}\;\text{of}\;\text{two}\;\text{levels}}{\text{one}-\text{half}\;\text{the}\;\text{difference}\;\text{of}\;\text{the}\;\text{levels}}$$

Actual experiments were performed in triplicates. The average of three experiments was compared with the predicted experiment. There was no significant difference observed between predicted and experimental values. The optimized experimental conditions in comparison to predicted experiment are depicted in Table 2.

Further, when drug was refluxed for 1 h with 0.5 N NaOH using heating mantle; around 8 % degradation was obtained. The time consumption for this was very high as compared to heating using microwave oven. Thus, it was proved that microwave energy can be used to expedite forced degradation of rebamipide under alkaline conditions.

### Development of Method

To get an adequate retention of rebamipide, initially, different mobile phases were tried in isocratic mode with a flow rate of 1 mL min^−1^. All the buffer solutions were prepared as per the procedure described by Snyder et al. [17]. Initially, acetonitrile: water (70: 30 %, v/v) mobile phase was tried. The retention time of rebamipide was 3.8 min. Further, acetonitrile: potassium phosphate buffer (pH 3, 0.02 M) (35: 65 %, v/v) was tried which gave a retention of 7.25 min. The same mobile phase was tried on the forced degradation samples, but rebamipide was found to be poorly resolved from its degradation products. Hence, it was decided to select the mobile phase which gives good resolution of rebamipide from its degradation products. The approximate similar type of result was obtained when acetate buffer was replaced with phosphate buffer. Now further, it was decided to increase the pH of the mobile phase. It has been found that mobile phase consisting of pH 6.8 buffers showed better resolution of the drug from its degradation products than those consisting of pH 3 buffers. Since the phosphate buffer has a better buffer capacity compared to acetate buffer, it was decided to select it as aqueous phase and methanol as organic phase. Hence, the mobile phase consisting of methanol: 0.02 M phosphate buffer (pH adjusted to 6.8) with the ratio of 60:40 % *v/v* was selected as the optimum mobile phase which gave good resolution of rebamipide from its degradation products along with good peak shape and lesser retention time. The maximum absorption wavelength of reference drug and forced degradation samples was found to be 230 nm and hence selected as detection wavelength for analysis.

**Fig. 3.**
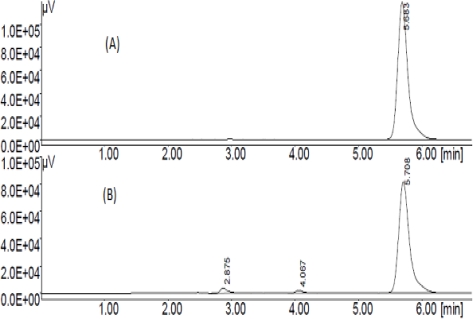
Chromatograms obtained from (A) Reference substance, (B) microwave assisted alkaline degradation

The average retention time was found to be 5.7 min. For alkaline stressed sample two degradation products were obtained one at 2.8 min (Deg 1) and another at 4.0 min (Deg 2), respectively. The chromatograms of unstressed rebamipide and alkali stressed samples of rebamipide are shown in Fig. 3.

### Method Validation

The data obtained in the calibration experiments when subjected to linear regression analysis showed a linear relationship between peak areas and concentrations in the range of 0.5–5 μg mL^−1^ (equation: Y = 601211X + 41127). When the tablets were analyzed using the developed method, the results obtained were in good agreement with the nominal amount of the drug. The drug content was found to be 101.39 ± 1.37 (n=3).

The data obtained from accuracy and precision experiments are summarized in Table 3.

The mean values of amount estimated of the drug were found very close to amount added and % RSD values of intra-day were found very low indicating acceptable accuracy and precision of the method. The intra and inter-day results at each level were subjected to one way ANOVA and F values at each level were obtained as a ratio of Between Mean Square to the Within Mean Square (F = BMS/WMS). The obtained values were found to be less than the tabulated F _(2, 6)_ at α = 0.05 (Tabulated F value = 5.14). These indicated that there was no significant difference between intra and inter-day variability, suggesting good intermediate precision of the method.

The chromatograms of blank and placebo solutions showed no interfering peak at the retention time of the drug indicating specificity of the developed method.

The method comply with system suitability each time in accordance to theoretical plates (more than 5000), tailing (below 2) and % RSD of the drug concentration in reference standard (n = 3) and test (n = 5) (below 2).

There was no significant change in retention time of rebamipide and its degradation products after introducing small changes in the mobile phase composition indicated robustness of the method.

### Forced degradation Behavior

**Fig. 4.**
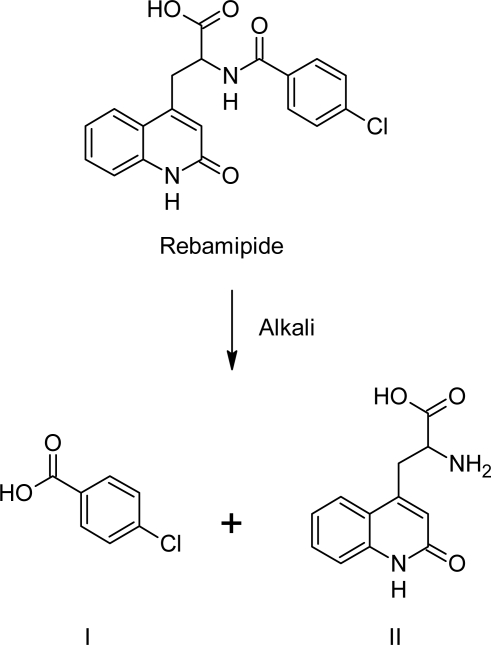
Proposed alkaline degradation pathway of rebamipide

The similar degradation behavior of the drug in bulk and tablets indicted that there was no interaction between the drug and excipients. The drug was found to be stable to acid hydrolysis, oxidative, wet heat conditions and photolytic conditions; as no decrease in peak area of drug was observed with no secondary peaks. The alkaline hydrolysis leads to breakdown of amide (C–N) bond which leads to the formation of two degradation products; *p*-chlorobenzoic acid (**I**) and 3-(2-oxo-1,2-dihydroquinolin-4-yl)alanine (**II**). When standard *p*-chlorobenzoic acid was chromatographed with same chromatographic conditions; it has been observed that retention time of Deg 2 matched with retention time of standard *p*-chloro benzoic acid (4.06 min for Deg 2 and 4.12 min for standard *p*-chlorobenzoic acid). Hence, it has been confirmed that one of the degradation product was *p*-chlorobenzoic acid (Deg 2) and another might be 3-(2-oxo-1,2-dihydroquinolin-4-yl)alanine (Deg 1) as shown in Fig. 4.

## Conclusion

A LC method was developed and validated statistically for quantitative determination of rebamipide. By making the use of full factorial experimental design real experimental situation was obtained which helped to reduce the trial and error experimentation. With Yate’s analysis the factor or combination of the factors which are most likely to effects degradation were identified. The comparison of conventional degradation using reflux and microwave assisted degradation proved that microwave energy can be utilized to expedite forced degradation for hydrolytic conditions. Validation experiments evidenced that the LC analytical method is linear in the proposed working range, as well as accurate, precise, specific and capable of separating the main drug from its degradation products. Due to these characteristics, the method is stability-indicating and may be applicable to routine analysis of rebamipide in tablets because there is no official method available.

## Figures and Tables

**Tab. 1.** Experimental matrix and their resulting % degradation in microwave assisted alkali hydrolysis

**Expt No.**	**X1**	**X2**	**X3**	**Degradation (%)**
1	0.1	15	420	0
2	1	15	420	0
3	0.1	30	420	2
4	1	30	420	8
5	0.1	15	700	17
6	1	15	700	21
7	0.1	30	700	28
8	1	30	700	33

X1: Concentration of NaOH (N), X2: irradiation time (min), X3: Microwave power (Watt)

**Tab. 2.** Optimized Experiment with comparison of Predicted and Observed results of alkaline condition

**X1 (Concentration of NaOH)**	**X2 (irradiation Time)**	**X3 (Microwave Power)**	**Predicted Degradation**	Obtained Degradation*
0.55 N	6.7 min	525 W	10%	8.74 ± 0.38 %

* Mean of three experiments ± SD

**Tab. 3.** Accuracy and Precision Studies

**Amount added (mg)**	**Amount found (mg)**			**Within mean square**	**Between mean square**	**F**

	**Days**					
	**1**	**2**	**3**			
	78.34	79.24	80.39			
**80**	79.43	81.09	80.43	1.64	1.72	1.05
	81.33	80.11	82.72			
**Mean**	**79.70**	**80.14**	**81.18**			
**% R. S. D.**	**1.89**	**1.15**	**1.64**			

	101.69	98.75	99.85			
**100**	99.68	97.83	102.79	2.79	6.79	2.43
	100.72	101.06	104.03			
**Mean**	**100.69**	**99.21**	**102.22**			
**% R. S. D.**	**0.99**	**1.67**	**2.10**			

	118.11	120.16	119.86			
**120**	120.36	119.88	120.43	2.34	3.82	1.63
	119.62	117.44	123.34			
**Mean**	**119.36**	**119.16**	**121.21**			
**% R. S. D.**	**0.96**	**1.25**	**1.53**			
